# Determination of the absolute stereostructure of a cyclic azobenzene from the crystal structure of the precursor containing a heavy element

**DOI:** 10.3762/bjoc.12.212

**Published:** 2016-10-19

**Authors:** Reji Thomas, Nobuyuki Tamaoki

**Affiliations:** 1 Research Institute for Electronic for Science, Hokkaido University, Kita-ku, N 20, W 10, Sapporo-001-0020, Japan; 2 Postgraduate and Research Department of Chemistry, Farook College, Farook College P. O., Kozhikode, 673632, Kerala, India

**Keywords:** azobenzene, cyclic compound, enantiomer, stereostructure, X-ray crystal analysis

## Abstract

Single crystal X-ray diffraction has been used as one of the common methods for the unambiguous determination of the absolute stereostructure of chiral molecules. However, this method is limited to molecules containing heavy atoms or to molecules with the possibility of functionalization with heavy elements or chiral internal references. Herein, we report the determination of the absolute stereostructure of the enantiomers of molecule (*E*)-**2**, which lacks the possibility of functionalization, using a reverse method, i.e., defunctionalization of its precursor of known stereostructure with bromine substitution (*S*-(−)-(*E*)-**1**). A reductive debromination of *S*-(−)-(*E*)-**1** results in formation of one of the enantiomers of (*E*)-**2**. Using a combination of HPLC and CD spectroscopy we could safely assign the stereostructure of one of the enantiomers of (*E*)-**2**, the reduced product *R*-(−)-(*E*)-**1**.

## Introduction

Chirality is a topic of fundamental importance in several branches of science [1–5]. Homochirality in nature was one of the most important challenges for researchers and the origin is still unsolved [6–8]. It is well known that biological functions of most living systems are determined by the stereostructure of biologically active molecules [9]. Beyond the academic interest, the synthesis and separation of chiral molecules plays a key role in chemical industry, particularly as catalysts [10] and pharmaceutical ingredients [11]. In addition to the special interest in molecular chirality in diverse fields of biology and pharmaceutical chemistry, several chiral molecules have been synthesised and studied for their stereostructure-dependent physical properties such as optical activity [12–22] and magnetic properties [23–25]. Hence, the chiral separation and the determination of absolute stereostructure are important aspects of stereochemistry for the complete understanding of the role of chiral structures in various physical and biological functions.

Until date there have been several spectroscopic, diffraction methods developed to determine the absolute stereostructure of chiral molecules [26–35]. The non-empirical methods employed to determine the absolute configuration of a molecule include circular dichroism, exciton chirality methods [28–29], NMR spectroscopy [30–32], X-ray diffraction [33–35], etc. In addition to these methods, many researchers explored a combination of vibrational circular dichroism and quantum mechanical calculations to determine the absolute stereostructures [36–38]. Among various methods used for determining the absolute stereostructure of the molecules, the X-ray diffraction methods obtained more credibility, especially the method using a single parameter viz. ‘Flack Parameter’ [33–35] which clearly identifies the absolute structure rather than measuring the intensity of the Bijvoet pairs [39]. However, the requirement of heavy atoms for the better anomalous dispersion, limits the usage of X-ray diffraction in determining the absolute stereostructure. In many of the molecules, the requirement of a heavy atom leads either to functionalization of the molecules with groups containing an heavy atom [40–44] or the relative position of the chiral centre is estimated in relation to an internal reference of known chirality [26]. Nevertheless, this approach can be applicable only to the molecules with easily functionalizable groups. Hence, it is important to provide an alternative method for the determination of the absolute stereostructure of a molecule without the possibility of functionalization.

Recently our group reported the synthesis of several cyclic azobenzene molecules and their properties [45–50]. All these molecules share a comparable cyclic structure, with a photoisomerizable azobenzene unit linked to a substituted or unsubstituted aromatic unit such as naphthalene or benzene. Generally the molecules are of three categories (i) achiral (irrespective of the isomerized state of the azobenzene, i.e., *E* or *Z*), (ii) chiral in *E*-state and achiral in *Z*-state of azobenzene, (iii) chiral in both states of the azobenzene unit. We have explored the property of planar chirality of these molecules and carried out the optical resolution to demonstrate various properties such as molecular brakes, chiroptical switches and chirality sensors for light and solvent etc. [45–50]. Although we could separate the enantiomers of all these chiral cyclic azobenzenes, the experimental determination of the absolute stereostructure by X-ray diffraction was difficult due to the lack of heavy elements.

In the present study, we demonstrate the determination of the absolute stereostructure of a molecule without any functional groups for the introduction of heavy atoms. We used a reverse reaction, i.e., not a reaction to add a heavy atom auxiliary to the chiral cyclic azobenzene but a reaction to remove the heavy atom from the precursor molecule with known chirality to obtain the target molecule. We could determine the stereostructure of the cyclic azobenzene by comparing the HPLC data obtained with a chiral column and the circular dichroism (CD) spectra of the product obtained by reduction of the enantiopure precursor and the target molecule.

## Results and Discussion

**Figure 1:**
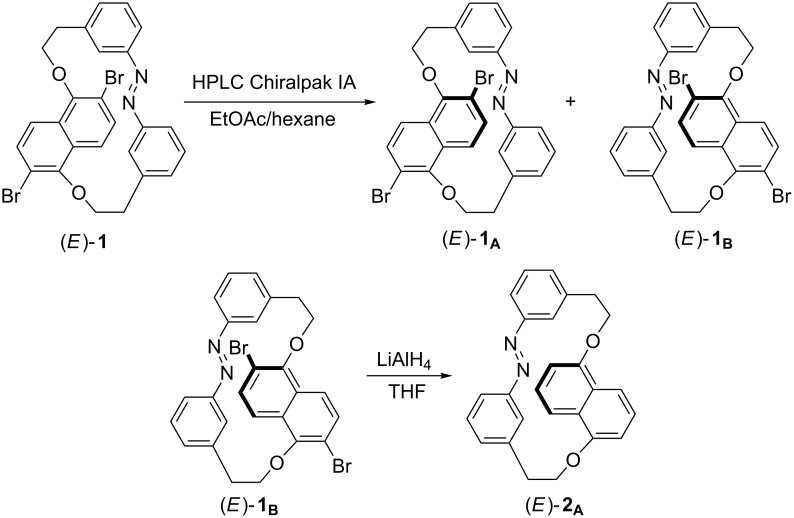
Schematic representation of the steps to enantiomer (*E*)-**2_A_**.

Figure 1 shows the schematic representation of experiments involved in the separation and reductive debromination of the enantiomer (*E*)-**1_B_**. The enantiomers of molecules (*E*)-**1** and (*E*)-**2** are previously reported as photocontrolled chiroptical switches for various nematic liquid crystalline hosts for tuning the reflection colors through the entire visible region [45–46]. The molecule (*E*)-**1** has been also used to demonstrate the completely photocontrolled rotation of the micro glass rods on the chiral nematic liquid crystalline films induced by the rotational reorganization of the polygonal finger print texture [45]. Molecule (*E*)-**1** contains a 2,6-dibromo-1,5-dihydroxynaphthalene part linked to an azobenzene unit through bismethylene spacers. The constricted rotation of the naphthalene unit in the cyclic structure gives planar chirality to this molecule with separable enantiomers. The presence of the bromine substitution in the naphthalene unit gives an added advantage in determining the absolute stereostructure of the separated enantiomers. The determination of the absolute stereostructure of one of the enantiomers of (*E*)-**1** by X-ray diffraction has been presented in our previous report [45]. In this study, we employ this enantiomer as a precursor for the determination of the absolute configuration of its reduced product, which is expected as one of the enantiomers of (*E*)-**2**.

**Figure 2:**
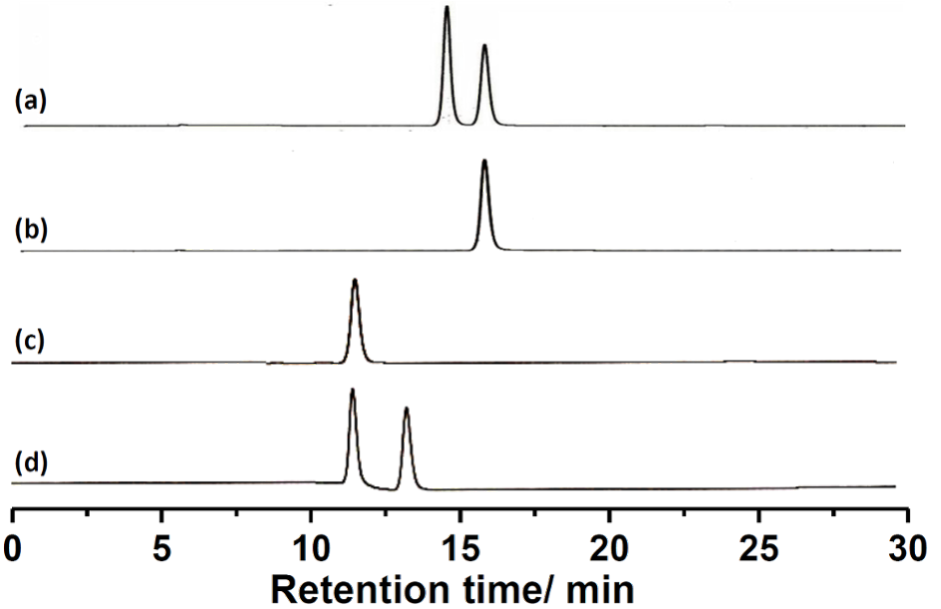
Chromatogram (Chiral HPLC) obtained for (a) racemic mixture of (*E*)-**1** (b) sample used for reductive debromination (second eluted enantiomer S-(−)-(*E*)-**1)** (c) recorded after the LiAlH_4_ reduction of S-(−)-(*E*)-**1** and (d) the chromatogram of racemic (*E*)-**2**. (Column: Chiralpak IA, eluent: ethyl acetate/hexane (10:90), Flow rate: 3 mL/min)

In order to characterize the reduced product we have carried out the ^1^H NMR spectroscopy of the compound and found that the spectrum completely matches with the previously reported compound (*E*)-**2** [46]. To confirm the formation of compound (*E*)-**2** we have carried out chiral HPLC studies on a Chiralpak IA column using a mixture of ethyl acetate and hexane (10:90) as the eluent to characterize the nature of the enantiomer formed by the reductive debromination. In the previous study with the combination of density functional theoretical calculations (DFT) and electronic circular dichroism (ECD), we have assigned the first and second eluted enantiomers of (*E*)-**2** as *R*-(−)-(*E*)-**2** and *S*-(+)-(*E*)-**2**, respectively, on a chiralpak IA column using hexane and ethyl acetate as eluent [46]. Figure 2a shows the chromatogram obtained for the racemic (*E*)-**1** with two peaks corresponding to the first (14.6 min) and second (15.9 min) eluted *trans* enantiomers, *R*-(+)-(*E*)-**1** and *S*-(−)-(*E*)-**1**, respectively. Whereas, Figure 2b shows the chromatogram recorded for the crystalline sample of *S*-(−)-(*E*)-**1** which is used for the reductive debromination. The chromatogram shows a single peak at 15.9 min ascertaining the enantiomeric purity of the sample. After reduction of *S*-(−)-(*E*)-**1** we have carried out the HPLC of the reduced sample where the chromatogram shows a single peak with a retention time of 11.9 min (Figure 2c). In order to obtain an idea about the enantiomeric nature of the reduced product we have recorded the chromatogram for the pure racemic sample of (*E*)-**2.** The chromatogram shows two peaks corresponding to first and second eluted enantiomers, respectively with the retention time of 11.9 and 13.5 min (Figure 2d). Comparing the chromatograms of the reduced product and the racemic mixture, it is clear that the reduced product is the first eluted enantiomer of (*E*)-**2**.

**Figure 3:**
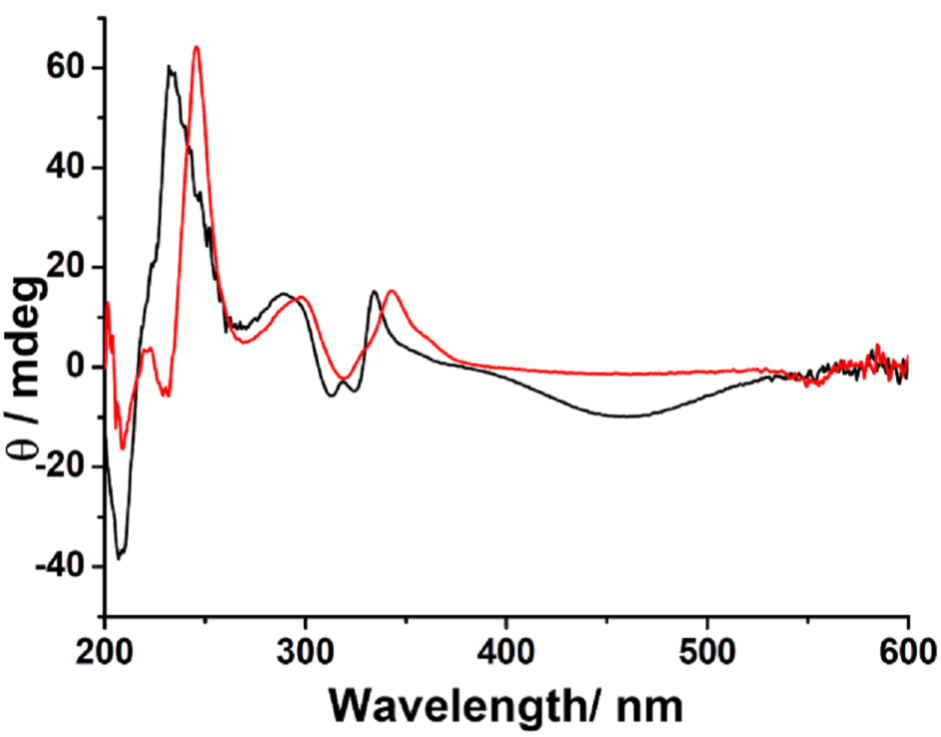
CD spectra recorded for solutions in THF for *S*-(−)-(*E*)-**1** (red curve) and for the reduced product of *S*-(−)-*E*-**1**, i.e., *R*-(−)-(*E*)-**2** (black curve).

To further confirm the formation of (*E*)-**2_A_** we studied the chiroptical properties of the product obtained by reductive debromination. Figure 3 shows the circular dichroism spectra of the reduced product along with that of *S*-(−)-(*E*)-**1**. From the spectral features and band intensity ratios, it is clear that the reduced product shows completely different cotton bands with that of *S*-(−)-(*E*)-**1**. We compared the spectra of the reduced product with that of the previously reported spectra of the enantiomers of (*E*)-**2** and found that the spectra matches the CD spectra of (*E*)-**2_A_** [46]. Thus from the HPLC and CD data it is clear that the reduced product of *S*-(−)-(*E*)-**1** gives the first eluted enantiomer of (*E*)-**2**.

According to the Cahn–Ingold–Prelog (CIP) priority rules [51], the absolute configuration of the reduced product should be ‘*R*’. The priorities of the functional groups on the reduced product reverse on removal of the bromine atom from the parent structure. Comparing the HPLC and CD data with structural data of *S*-(−)-(*E*)-**1** the absolute stereostructure of its reduced product is determined as *R*-(−)-(*E*)-**2**. The experimentally derived stereostructure completely matches with the absolute structure previously predicted from theoretical calculations.

In summary, we demonstrated the determination of the absolute configuration of the enantiomers of a molecule from its precursor with known stereostructure. More importantly, this method can be used for the determination of stereostructures of molecules without functional groups for the functionalization with heavy elements or chiral internal references.

## Experimental

All solvents and chemicals were obtained from commercial sources and used without further purification, unless otherwise stated. NMR (^1^H and ^13^C) spectra were recorded with a JEOL ECX 400 spectrometer using tetramethylsilane as an internal standard. Matrix-assisted laser desorption ionization time-of-flight mass spectrometry (MALDI–TOFMS) was performed with an Applied Biosystems Voyager-DE pro instrument. Absorption spectra were recorded with an Agilent 8453 spectrophotometer and the CD spectra were recorded with a JASCO J-S720 CD spectrophotometer. High-performance liquid chromatography (HPLC) was conducted with a Hitachi Elite La Chrome HPLC system using CHIRALPAK IA (DAICEL Chemical Industries Ltd.) column with solvent mixtures of ethyl acetate in hexane, 10:90 as eluent for the HPLC experiments at a flow rate of 3 mL/min.

### 

#### Reductive debromination of compound (*E*)-**1_B_**

To a solution of freshly separated enantiomer (*E*)**-1_B_** (30 mg, 0.054 mmol) in THF, LiAlH_4_ (20.49 mg, 0.54 mmol) in THF was added in drops at room temperature and stirred overnight. The reaction mixture was carefully quenched by adding water dropwise and the reaction mixture was filtered to remove the precipitate. The filtrate was separated with ethyl acetate, evaporated and column chromatographed over silica gel using a mixture of ethyl acetate in hexane (10:90) as eluent to obtain the debrominated product (yield: 80%). ^1^H NMR (400 MHz, CDCl_3_, 25 °C, TMS) δ 7.67 (d, *J* = 8.8 Hz, 2H), 7.55 (d, *J* = 8.4 Hz, 2H), 7.35 (t, *J* = 7.6 Hz, 2H), 7.23 (d, *J* = 6.8 Hz, 2H), 7.05 (t, *J* = 8.0 Hz, 2H), 6.79 (s, 2H), 6.60 (d, *J* = 7.6 Hz, 2H), 4.76 (m, *J* = 14.6 Hz, 2H), 4.60 (m, 2H), 3.21 (dd, *J* = 3.2, 4.8 Hz, 4H); ^13^C NMR (75 MHz, CDCl_3_) δ 154.1, 152.3, 140.4, 130.6, 128.9, 127.9, 125.6, 125.0, 120.0, 114.6, 107.7, 68.4, 35.9.
